# Exploring Undergraduate Nursing Students' Understandings of Mental Health: From Fear and Stigma to Holistic Understanding

**DOI:** 10.1111/inm.70135

**Published:** 2025-09-04

**Authors:** Sinead Barry, Louise Ward, Ruby Walter

**Affiliations:** ^1^ RMIT University Melbourne Victoria Australia; ^2^ Southern Cross University Lismore New South Wales Australia

**Keywords:** mental health, nursing, phenomenography, student, understanding

## Abstract

This research explored the variation in undergraduate nursing students' understandings of mental health (MH) at a Melbourne metropolitan university. Using phenomenography, a qualitative research methodology, the study involved interviewing 19 students at different stages of a 3‐year Bachelor of Nursing or Bachelor of Nursing and Midwifery degree. The research was undertaken as part of a PhD study undertaken between 2016 and 2020. The findings revealed seven distinct categories of understanding of MH, ranging from fear‐driven misconceptions of mental health disorder (MHD) to a holistic perspective recognising MH on a spectrum potentially fluctuating throughout an individual's lifespan. This research revealed an improvement in nursing students' understanding of MH with initial responses marked by fear and anxiety rooted in cultural beliefs and personal safety concerns. As understanding developed, students focused on physical symptoms and causality, sometimes attributing MHDs to personal misfortune or choice. The most advanced level demonstrated a sophisticated, integrated view recognising the interconnectedness of mental and physical health. These findings highlight the challenges students face in understanding MH and the critical need to address misconceptions within nursing curricula. The study recognises the importance of innovative educational strategies to enhance MH literacy among nursing students. The findings contribute valuable insights into teaching practices and the development of targeted educational interventions in MH nursing education.

The landscape of nursing education has increasingly recognised the importance of integrating comprehensive MH content to prepare students for the complexities of modern healthcare. Despite this recognition, disparities in education persist, posing challenges for undergraduate nursing students in developing a holistic understanding of MH (Lakeman et al. 2024). Recent studies have identified a significant gap in the literature regarding nursing students' perceptions of MH, particularly in how these perceptions change and develop throughout their education (Ward and Barry 2018).

A recurring theme within nursing literature is the fear and stigma associated with MH and mental health disorder (MHD) among nursing students. Fear often stems from cultural beliefs and concerns about personal safety, hindering students' ability to engage fully with MH content and to consider careers in MH nursing (Garvey et al. 2021; Happell et al. 2019). Despite the increasing demand for MH services within the healthcare system, MH nursing continues to be a less preferred career option among nursing graduates (Ong et al. 2017). Therefore, addressing these misconceptions is crucial to encourage more students to pursue careers in this essential field.

Exploring students' understanding of MH is crucial for nursing education (Barry and Ward 2017). Defining MH as distinct from MH conditions remains elusive, with studies showing university students often conflate MHD and MH, using the terms interchangeably (Jithoo 2018). Research across various disciplines has found confusion and a tendency to describe MH in negative, illness‐focused terms (Laidlaw et al. 2016). Rodwell et al. (2017) found that attitudes and stigma around MH and MHDs develop early, before students fully grasp these concepts. There is a current gap in research exploring MH understanding from a student nurse perspective. This study uses phenomenography to investigate undergraduate nursing students' perceptions of MH at a Melbourne metropolitan university, providing insights to inform and enhance MH nursing education.

## 1 Background

Since the introduction of comprehensive nursing education in Australia in the 1980s, inadequate MH nursing content in pre‐registration curricula has been a persistent concern (Lakeman et al. 2024). Despite ongoing debate and government inquiries, students continue to feel fearful (Hunter et al. 2015), anxious (Thongpriwan et al. 2015) and unprepared (Koskinen et al. 2011) for MH nursing. Efforts by professional bodies and leaders have not resolved this issue (Lakeman et al. 2024). The comprehensive model's limitations in preparing graduates for MH roles remain widely debated, with calls for reform to better meet workforce needs (Lakeman et al. 2024).

The quality and quantity of MH nursing education in Australia varies widely across programmes. Happell (2009) found that more MH nursing hours led to improved student attitudes and workforce readiness. However, MH nursing often provokes fear and anxiety (Garvey et al. 2021; Hunter et al. 2015; Thongpriwan et al. 2015), contributing to its low selection by graduates (Ong et al. 2017). Addressing these issues in curricula is essential to better support student learning in MH.

Australian literature highlights nursing students often experience heightened anxiety and fear in MH clinical settings, largely due to concerns about violence and unpredictable behaviour associated with MHDs (Goh et al. 2021; Ward and Barry 2018). Heightened anxiety also corresponds with an increased sense of fear for personal safety whilst in the MH clinical setting (Yildiz 2021). This fear can hinder learning and deter students from pursuing MH nursing careers (Happell 2008). However, innovative pedagogies like simulation have been shown to build student confidence in communication skills and preparedness for practice (Garvey et al. 2021; Goh et al. 2021).

Integrating virtual reality (VR) based simulation into MH nursing education has shown promising results. Used as a complement to clinical practice, VR promotes a risk‐free environment that replicates MH clinical settings, enhancing students' preparedness, communication and risk management skills (Lee et al. 2024). The reassurance of safety is an integral part of supporting student learning, especially since students' perception of fear and safety dominates literature within MH nursing education.

Integrating consumer‐oriented lived experience practices, such as expert by experience (EbE) involvement, enhances MH nursing education by supporting recovery‐oriented practice and fostering positive attitudinal shifts (Happell et al. 2019). Embedding EbE throughout the curriculum, rather than in standalone subjects, leads to deeper understanding (Happell et al. 2019). Co‐production with EbE strengthens students' humanistic views and helps them see beyond the illness (Horgan et al. 2021), transforming MH nursing education. With 50% of hospital admissions involving comorbid physical and MHD presentations (Barrett and Jackson 2013), nursing graduates must be prepared to support these patients. Understanding students' perceptions of MH can inform improvements in teaching and learning strategies, enhancing MH care nationally and internationally.

## 2 Methods

### 2.1 Design

Phenomenography was used to explore the variation in undergraduate nursing students' understandings of MH. Phenomenography is designed to ‘map the qualitatively different ways people experience, conceptualise, perceive, and understand various aspects of, and the phenomena in, the world around them’ (Marton 1986, 31). It is recognised as a valuable approach in nursing education to improve teaching and learning principles (McClenny 2020). The methodology has previously led to new discoveries into understanding learners' experiences of complex and challenging areas of knowledge and the differing experiences of a nursing phenomenon (McClenny 2020).

This study was conducted at a large metropolitan university in Melbourne, Victoria and Australia. First‐, second‐ and third‐year students enrolled in either the Bachelor of Nursing (BN) or Bachelor of Nursing and Midwifery (BNBM) degree were invited to participate in the study. Following ethics approval, 19 participants consented to participate in semi‐structured interviews.

### 2.2 Data Collection

Phenomenography uses individual semi‐structured interviews for data collection (Larsson and Holmström 2007). Interview questions, developed according to phenomenographic principles to explore varied participant experiences (Sims 2024), were asked in a consistent sequence. The guide combined pre‐determined, open‐ended questions with flexible prompts to encourage in‐depth responses. Participants were recruited via email through an independent officer, with snowball sampling used as needed. Face to face interviews were held in a private room, lasted 45 to 60 min, were audio recorded and transcribed verbatim. All interviews were conducted by an academic researcher who is also a MH nurse.

Table 1 illustrates the two core questions and five follow‐up probing questions that each of the 19 participants was asked.

**TABLE 1:** Interview questions.

What do you think MH is?
Is there an experience that stood out and influenced your opinion?
Is there anything outside of university that perhaps shaped your opinion of what MH means to you?
Thinking back on your time at university as an undergraduate nursing student how do you think your views of MH have changed?
What was your view when you started and how has it changed?
On your first day of nursing what did you think about MH?
How has your understanding of MH shifted since your first day of nursing?

### 2.3 Ethics

The project received approval as an ‘above low risk’ application from the University Human Research Ethics Committee (HREC), ensuring compliance with national and university ethical standards. This classification was due to the sensitive nature of discussing students' understandings of MH and the potential for personal disclosures. Interested students responded to the recruitment email and completed the participant information and consent form.

### 2.4 Analysis

**FIGURE 1:**
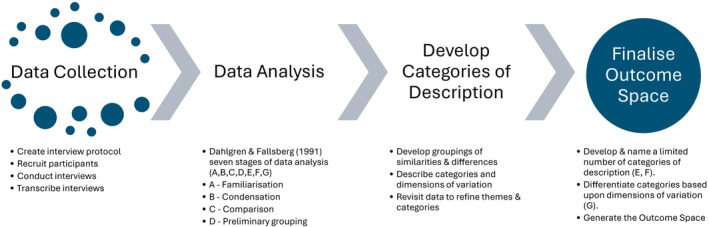
Phenomenographic process and data analysis.

Phenomenographic data analysis relies solely on interview transcripts as evidence (Green 2005). Themes are discovered inductively as the researcher immerses themselves in the transcripts through repeated reading to become thoroughly familiar with the content (Sjöström and Dahlgren 2002). This process leads to the emergence of initial categories that capture the qualitatively different ways students understood MH. The goal is to develop categories of description (CoD), each representing a distinct variation in understanding that is logically connected to others and arranged hierarchically (Sims 2024). Figure 1 provides an overview of the phenomenographic process of data collection and analysis.

Qualitative research rigour and trustworthiness were maintained by using reflexive journaling to ensure unbiased data collection and analysis (Sims 2024). The researcher consciously reflected on their thoughts, feelings and assumptions—especially those shaped by prior MH nursing education research—to recognise and manage potential biases throughout the study.

## 3 Results

**FIGURE 2:**
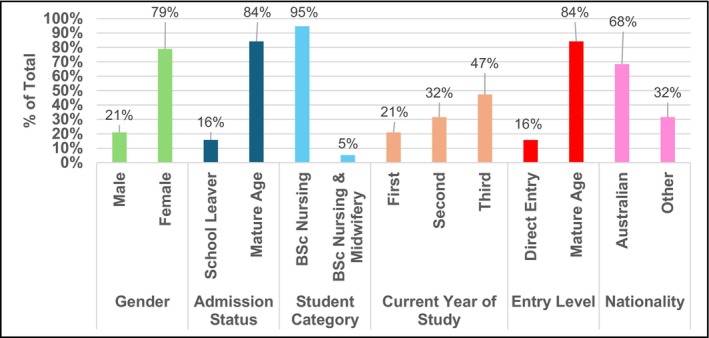
Participant demographics.

Recruiting participants to achieve maximum sample variation was essential to reflect the demographic diversity of students enrolled in the undergraduate nursing degree. Data saturation was reached after 19 interviews, as no new variations emerged and the demographic diversity of participants was well represented. Demographic diversity was considered a second‐level component of saturation, with efforts made to include a range of backgrounds reflective of the university's student profile. This approach aligns with phenomenography's aim to capture variation in how a phenomenon is understood, making it appropriate to recruit participants mirroring the diversity of this large metropolitan university. The participant diversity is illustrated in Figure 2.

Figure 2 shows the demographics of the 19 nursing student participants: 15 females and four males, spanning all 3 years of the BN course and including both school leavers and mature‐age students. Most entered nursing directly, with a few receiving advanced standing, and the sample reflects the typical gender and enrolment patterns of the programme, with 18 in the BN and one in the BNBM.

**FIGURE 3:**
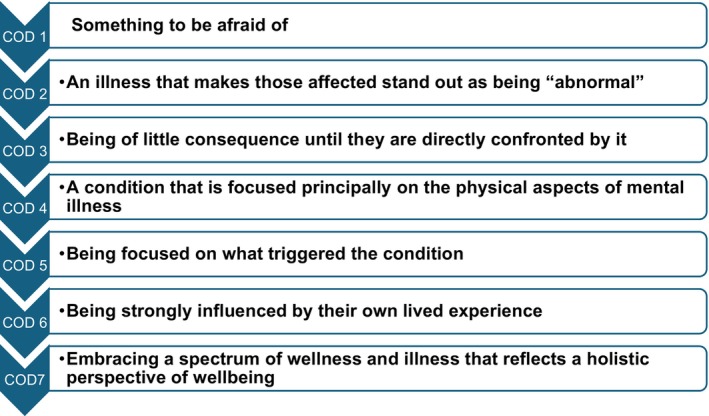
The outcome space representing the variation in undergraduate nursing students' understandings of MH.

Figure 3 presents the Outcome Space, illustrating the seven distinct and increasingly complex ways nursing students understood MH, from partial understandings at CoD 1 to more comprehensive views at CoD 7.

### 3.1 CoD 1: Nursing Students Understanding That MH Is Something to Be Afraid of

This category reflects students whose understanding of MH was shaped by fear, primarily associated with MHDs. Despite being asked about MH, their responses focused on MHD, expressing concerns for personal safety and using terms like ‘crazy’, ‘violent’, ‘aggressive’, ‘scary’, and ‘possessed by the devil’. Participant 10 specifically mentioned fear when encountering someone with a MHD.

Well, when I was quite young it was quite scary ‘cause I had one friend whose brother was severely mentally ill and aggressive and stuff, so that probably was quite scary, I think. He was really unwell and undiagnosed at that early stage and quite violent as well, especially towards his brother who was my friend, so that was a scary experience.

The use of stigmatised and discriminatory language further confirms the level of fear held by students relating to MHDs. Participant 8 explained:

I came here when I was eight, but even there now, when you hear some of our community members had committed suicide, you hear comments like “Oh, he's been possessed by the devil or whatever.” I had little things like that before you hear about it, and someone, like from your culture, thinks they don't believe in depression as mental illness at all, so it's different to what I used to hear before – like a women will go through post‐natal depression, and they will tell her she is crazy and she has lost the plot; yeah its different.

The students' behaviours were strongly influenced by their fear driven understanding of MH. Participant 9 described their attempt to actively avoid encounters with an individual with a MHD due to fear for their own personal safety.

Not very good, like I think there was some stigma in mind or discrimination. Like we were scared of mental health and basically it depends upon the culture as well. Like in India there is not much care taken about mental health, so you are scared of it and you want to stay away from a person with a mental illness.

### 3.2 CoD 2: Nursing Students Understanding That MH Is an Illness That Makes Those Affected Stand out as Being ‘Abnormal’

In this CoD nursing students' understandings are focused on identifying MHDs as an ‘abnormal’ state. Consistent with understandings in CoD 1, understandings in CoD 2 again focused on MHD rather than MH and build on the fear associated with MHD. Labelling and discrimination were used as a judgmental assessment that underpinned the understandings in this category. This is shown in Participant 8's description:

Someone that's not physically sick, but mentally. It's the way they talk – it doesn't sound right, or they have hallucinations or other things that are not really there.

The use of language such as ‘normal’ and ‘abnormal’ is an example of the stigmatised and discriminatory language used by students, as evident in Participant 15's response:

I guess a really “normal” [person] would probably be someone who doesn't have mental health illness.

Interestingly, students' understandings continue to interpret the term ‘mental’ with the understanding that it is an illness, rather than a state of health. A suggestion for this behaviour may relate to students considering the terms as one and the same. Participant 16 describes:

Mental health to me is being abnormal, not being able to cope with the functions of normal life – I suppose an illness.

Students overwhelmingly understand MH as a problem rather than an optimum state of health. Participant 17 summarises:

I just think that if they had told me about [their] mental health or mental condition, then I would have thought there was some problem. Mental health means they were some kind of psycho or like that is what I used to think

### 3.3 CoD 3: Nursing Students' Understanding That MH Is of Little Consequence Until They Are Directly Confronted by It

This third CoD depicts students' understandings of MH that are described as being of little consequence until they are directly confronted by it. There appears to be an awareness, albeit somewhat superficial, that MH is out there in a broader sense; however, understandings reflected students' unwillingness to consider the concept unless there is a personal need. Students' understandings in this category focus on rationalising their limited awareness of MH as seen in Participant 5's description:

I grew up in a rural town, so that is number one, I guess, (laugh) for not having had any contact with anyone who had a mental illness and had sought help, professional help.

Participant 8 also builds on the theme of exposure and describes their understanding of MH by assuming it would be overtly obvious to identify in individuals.

The thing is I am only exposed to it now as I haven't actually seen it before.

It is evident that MH has not been a concept in nursing students' field of vision.

P15‐ Well, I am not too sure. I have never really encountered anyone, but I do have a cousin that inherited it. I'm not too sure what he has, to be honest, but he has inherited it from his mum's side.

Participant 12, provides justification for their inability to explain MH:

We have not touched on mental health whatsoever in uni – we haven't even spoken about it. I mean obviously we have spoken about acquired brain injuries and the impact that they can have long term because some people have come across what's happening in ABI [acquired brain injury] wards on placements already, but not the broad sense of the mental health side of things.

### 3.4 CoD 4: Nursing Students Understanding That MH Is a Condition That Is Focused Principally on the Physical Aspects of Mental Illness

Nursing students' understandings within this CoD are predominantly focused on the physical aspects of MH, including separating MH from physical health. Consistent with earlier discussed categories, students were again focused on MHD, not MH; however, unique to this CoD was students understanding that MH was isolated to the brain. Participant 1 describes:

I think mental health is a condition that people in the community or throughout the world may have. It's usually in the brain and could have developed from things people had experienced or be a genetic condition they were born with.

The perceived separation between MH and physical health was again reinforced by Participant 17:

I think mental health refers to the brain and cognitive ability of an individual; it's like I am thinking and forming a new information memory and information of new ideas. They are all related to our mental capacity and what mental health means to me is how can we deal with our mental abilities; how can we learn new things.

Participant 3 also viewed MH as being separate from physical health.

But it's still really, really prevalent and also the idea it's still completely separate from physical health and that you know they exist separately

Participant 3 provided recommendations suggesting a greater (or perhaps more visible) proportion of nursing curriculum content should be on physical and MH stating:

I think having that [mental health] expressed in the nursing curriculum would be really beneficial … and when you are talking about something with such high prevalence it's ridiculous that we talk about it being separate. I would like to imagine it could be a bit more incorporated in future and that would reduce the stigma, the fear and the other associations.

### 3.5 CoD 5: Nursing Students Understanding That MH Is Focused on What Triggered the Condition

Category 5 reflected students' understandings of MH that were influenced by judgemental assessments of causality. This is depicted in Participant 4's reflection:

I used to have the thought that mental health was always self‐inflicted somewhere along the line; it didn't just happen. It's been caused by someone's actions, whether by them or those around them. I didn't realise that sometimes that is just how life is, or how the brain works, or the environments that you are in. I now realise that mental health is not always someone's fault – like things just happen.

Participant 18 reflected upon their family upbringing and how this influenced their understanding that there was always a reason for MI.

My parents would say there would always be a reason – like you would see people on the news who would be described as sick… There would always be a stigma, a title – “they're on a bad road” – so, yeah, they're mental. We discussed it, but Mum and Dad always had a reason for why people were ill, or why they had a mental condition. It was just that it wouldn't happen in our family; but it did happen in our family because my grandmother was sick. But it happened to me because I had a mental … I had a road accident. It also happened to my brother because he met the wrong friends, but my parents didn't really see that it could affect anybody.

Consistent with all previously discussed CoD, MH is continuing to be misunderstood as a MHD. However, a key distinction is that students understand mental health by apportioning blame. This was evident from Participant 4's responses:

I used to have the thought that mental health was always self‐inflicted somewhere along the line; it didn't just happen. It's been caused by someone's actions, whether by them or those around them.

### 3.6 CoD 6: Nursing Students Understanding That MH Is Strongly Influenced by Their Own Lived Experience

In the sixth CoD, nursing students used their lived experience to inform their understanding of MH, but often still conflated MH with MHDs. They recognised that familiarity with MHDs offered only partial insight, as Participant 12 noted there was still much to learn despite personal experience.

I am thankful I have had experiences like that. But then in saying that, what I know of would only scrape the surface; there is still so, so much to learn.

There was however a belief that to understand MHDs one must have experienced a MHD. This was reflected by Participant 7:

It makes me angry; it's not funny at all, and I know that people won't understand it unless they have had it.

Participant 3 shared how their own understanding of MH had been transformed due to their previous experiences:

And then in the past five to 10 years, I guess, my own mental health has changed a bit so I have gained a greater understanding of what it's like to experience that and the treatment options and the responses you get from people.

Despite students demonstrating a greater awareness of MHDs than previously discussed CoD, understandings still fell short of explaining what students understood of the concept of MH.

### 3.7 CoD 7: Nursing Students' Understanding That MH Embraces a Spectrum of Wellness and Illness That Reflects a Holistic Perspective of Wellbeing

It was not until CoD 7 that students demonstrated a comprehensive understanding of MH, not MHDs. Understandings were focused on the multidimensional aspect of health, incorporating wellness, illness, physical, social and emotional components of health.

Students referenced MH being positioned on a spectrum of illness and wellness. This is evident with Participant 3's explanation:

I guess mental health is our internal state, so our mental state and mental health can be characterised by mental illness or wellness, or it's sort of a spectrum in between.

Participant 9's comment below, demonstrate a holistic approach to MH.

For me mental health is what I understand from my placements; it is holistic care. Like in other fields you have to stick to the physical health but in this you have to consider the mental, physical, social and emotional aspects.

As distinct from previously discussed CoD, there was a clear understanding that MH could not be separated from physical health. Despite referring to the term ‘normal’ Participant 3 describes their high‐level understanding with empathy, and no judgement between ‘them and us’ like the earlier CoD.

I am trying to sort of impress upon my fellow students a bit that it is just so normal; it's not separate from your life nor is it separate from reality. If you have a mental illness, you have to go to work and you may do further study, or whatever you have got to do to raise a family – all of that stuff. The mental health stuff just comes along with that and you just have to figure out how to make that work. It's all the normal stuff that needs to be incorporated somehow; it's about learning to manage and cope with it and fit that around what you want to be doing in your life.

## 4 Discussion

Nursing students' understandings of MH are influenced by stigma, cultural background, limited exposure and educational gaps, as seen in both Australian and international literature. Many students expressed anxiety and often linked MH with MHDs and negative stereotypes, leading to reluctance to engage with MH consumers (Goh et al. 2021; Ward and Barry 2018). Stigma remains a significant barrier, with some students labelling those with MH issues as ‘*abnormal*’ or ‘*psycho*’. Fear inhibits students' sense of professional connectedness in MH care (Sun et al. 2020) and contributes to reservations about working with MH consumers (Ward and Barry 2018). Anti‐stigma interventions, especially those involving direct contact or simulation, have been shown to reduce negative attitudes and increase willingness to care for people with MHDs (Sreeram et al. 2022).

Cultural influences were pronounced, as some students attributed MHDs to supernatural or moral causes, a trend also identified in the literature, particularly among students from diverse backgrounds (Rayan 2024). For example, nursing students referenced cultural influences from their upbringing, with comments such as, ‘…you will hear some of our community member [sic] committed suicide and like “oh he's possessed by the devil or whatever”…’. Suggestions of cultural influence were substantiated by Reavley et al. (2012), who concluded that students born outside of Australia were more likely to hold poorer MH literacy compared to their Australian‐born fellow students. Recognising the impact of cultural background on MH literacy is crucial for nursing education, particularly in diverse student populations (Hansen et al. 2021).

Limited awareness and recognition of MH were evident, especially among nursing students with little personal exposure or those in earlier years of study. Millar (2017) found that MH literacy improves with academic progression and personal proximity to MH issues, though significant gaps remain. Within this study, many students reported no prior exposure to MH or MHDs and highlighted their limited knowledge. These findings, along with Millar's (2017), support the need for curriculum development to address MH literacy gaps at all stages. Nursing education is crucial for developing MH literacy, but students face challenges in recognising MH within a broad curriculum. Education remains a powerful tool to improve MH literacy (Ward and Barry 2018), positioning nursing programmes to enhance students' understanding across all year levels.

A dualistic view separating MH from physical health was common, despite extensive evidence of the mind–body connection and increased morbidity among those with MHDs. Students often saw MH and physical health as separate entities, as reflected in comments like ‘…they exist separately…’ and ‘mental illness [is] as disease of the mind’. This lack of awareness about the mind–body connection is concerning, given the higher morbidity rates of chronic diseases among individuals with MHDs (Bartlem et al. 2015). This finding underscores the need for nursing education to make concerted efforts to integrate holistic health concepts.

The literature consistently shows that attributing blame for MHDs drives stigma and negative attitudes. Forde and O'Shea (2025) found that when MHDs are seen as resulting from personal failings, people are more likely to blame the individual. The current study aligns with Forde and O'Shea (2025) and demonstrates that blame attribution fosters stigma (Graham 2020). Comments from participants such as ‘I used to have the thought of mental health that it was always self‐inflicted somewhere along the line. It was self‐inflicted. It didn't just happen; it's been caused by someone's actions whether it would be them or those around them’, illustrate how focusing on causality can hinder understanding and prioritisation of MH care. Stereotyping remains a major barrier and nurses who view patients as responsible for their illness may show less empathy, affecting care quality. These findings underscore the need for education to address misconceptions about MHD causes and reduce blame‐based stigma among nursing students.

Personal or vicarious experience with MHDs enhances empathy and understanding, supporting evidence that contact‐based education and exposure to lived experience reduce bias and foster positive attitudes. Nearly half of Australians aged 16–85 have experienced a mental disorder (Australian Bureau of Statistics 2023), so familiarity is common. Students' remarks like, ‘…I have gained a greater understanding of what it's like to experience that [mental illness]…’ and ‘…had a few experiences with friends and family that have had mental health issues’… show how personal connections deepen understanding. This familiarity reduces bias and encourages positive attitudes (Zeng et al. 2024), highlighting the value of early and repeated MH care exposure in nursing curricula. Contact‐based education and lived experience, including MH simulation, are well established as effective in reducing bias and promoting positive attitudes among students (Alexander et al. 2023).

Some students demonstrated a sophisticated, holistic understanding of MH, recognising its inseparability from physical, social and emotional wellbeing—an approach aligned with the World Health Organisation's definition (World Health Organization 2024) and supported by literature showing that improved MH literacy enables comprehensive, person‐centred care. Students emphasised that MH cannot be separated from life, noting ‘it's kinda [sic] ridiculous that we talk about it being separate’ and ‘you need to have good mental health in order to have good physical health…. A holistic understanding of MH, one that acknowledges its inseparability from physical, social and emotional well‐being, is essential for effective, person‐centred nursing care. Improving nursing students MH literacy, especially when it is grounded in holistic models, enables nursing students to deliver truly comprehensive, person‐centred care.

This study contributes to the literature by providing rich qualitative insights into the nuanced ways nursing students conceptualise MH and MHDs, revealing the persistence of stigma, dualism and cultural misconceptions in their own words. It highlights the significance of cultural background, the persistence of gaps across year levels and the transformative role of personal experience in shaping attitudes. By advocating for targeted educational strategies—such as anti‐stigma interventions, integration of holistic health concepts and increased exposure to MH care—this research offers actionable recommendations to address persistent barriers and enhance MH literacy among nursing students, advancing the field's understanding of how best to prepare future nurses for comprehensive MH care.

### 4.1 Limitations

The results from this research are contextual to the students who participated in the study and were enrolled in either a BN or BNBM degree at one Melbourne metropolitan university. The results from this study are not intended to be generalised to all nursing students' understandings of MH. Rather, they are representative of the variation in understandings at a snapshot in time. Participation in the research was on a voluntary basis; therefore, there is potential that some students who had a personal interest in MH volunteered to participate. However, the variation in CoD presented in this study confirms there was a wide spread of understandings.

## 5 Conclusion

The aim of this research was to reveal the variation in students' understandings of MH. Findings uncovered seven differing ways of understanding MH and identified some of the key influential factors shaping student understandings. By exposing the variation in understandings of MH, it has the potential to act as an early intervention strategy to guide change in nursing education. The seven differing ways that MH is understood advance nursing knowledge in this area.

This research has shown the intricate nature of understanding MH and supports previous research identifying the challenges students face when embarking on MH nursing education. These findings have shown MH is a highly complex concept proving challenging for many nursing students to comprehend, regardless of previous theoretical, clinical, or personal exposure. Consequently, this provides valuable insight from a teaching and learning perspective of the challenges encountered by nursing students when considering a state of MH. The findings demonstrate that nursing education would be enhanced by a greater awareness of the way students understand MH and the influence that this may have on the quality of care.

Student understandings of MH were dominated by two factors: an illness focus and suggestions of stigma associated with MHDs, both with a negative effect. The knowledge gained from undertaking this research adds to the body of literature supporting the need for innovative and creative approaches to support student learning in MH education.

## 6 Relevance for Clinical Practice

Exposing challenges students encounter when understanding key concepts in healthcare provides a powerful opportunity for nursing education to act on and support student learning. There is a need to expose the variation in nursing students' understandings of MH to bring awareness that current educational methods fail to shift engrained, negative and stigmatised views associated with MHDs. Misunderstanding MH undoubtedly has an impact on the quality of care that is provided to individuals with a MHD.

## Ethics Statement

The authors have nothing to report.

## Consent

The authors have nothing to report.

## Conflicts of Interest

The authors declare no conflicts of interest.

## Data Availability

Research data are not shared.
